# Elevated transaminases and hypoalbuminemia in Covid-19 are prognostic factors for disease severity

**DOI:** 10.1038/s41598-021-89340-y

**Published:** 2021-05-13

**Authors:** Jason Wagner, Victor Garcia-Rodriguez, Abraham Yu, Barbara Dutra, Scott Larson, Brooks Cash, Andrew DuPont, Ahmad Farooq

**Affiliations:** 1 Department of Internal Medicine, University of Texas Health Science Center at Houston, 6431 Fannin, MSB 1.150, Houston, TX 77030 USA; 2 Department of Internal Medicine, Division of Gastroenterology, Hepatology and Nutrition, St. Joseph Medical Center, Steward Health, 1315 St Joseph Parkway, Houston, TX 77002 USA; 3 Department of Internal Medicine, Division of Gastroenterology and Hepatology, Duke University, Durham, NC USA

**Keywords:** Gastroenterology, Medical research

## Abstract

Prognostic markers are needed to understand the disease course and severity in patients with Covid-19. There is evidence that Covid-19 causes gastrointestinal symptoms and abnormalities in liver enzymes. We aimed to determine if hepatobiliary laboratory data could predict disease severity in patients with Covid-19. In this retrospective, single institution, cohort study that analyzed patients admitted to a community academic hospital with the diagnosis of Covid-19, we found that elevations of Aspartate Aminotransferase (AST), Alanine Aminotransferase (ALT) and Alkaline Phosphatase (AP) at any time during hospital admission increased the odds of ICU admission by 5.12 (95% CI: 1.55–16.89; *p* = 0.007), 4.71 (95% CI: 1.51–14.69; *p* = 0.01) and 4.12 (95% CI: 1.21–14.06, *p* = 0.02), respectively. Hypoalbuminemia found at the time of admission to the hospital was associated with increased mortality (*p* = 0.02), hypotension (*p* = 0.03), and need for vasopressors (*p* = 0.02), intubation (*p* = 0.01) and hemodialysis (*p* = 0.002). Additionally, there was evidence of liver injury: AST was significantly elevated above baseline in patients admitted to the ICU (54.2 ± 15.70 U/L) relative to those who were not (9.2 ± 4.89 U/L; *p* = 0.01). Taken together, this study found that hypoalbuminemia and abnormalities in hepatobiliary laboratory data may be prognostic factors for disease severity in patients admitted to the hospital with Covid-19.

## Introduction

Covid-19 is a respiratory illness caused by the SARS-CoV-2 virus^1^. Prognostic markers are needed to aid clinicians in understanding disease course and severity. There has been abundant clinical and biochemical evidence that SARS-CoV-2 infects the gastrointestinal and hepatobiliary tract^2,3^. Additionally, elevated transaminases have been frequently reported during the initial stages of the disease^1,4,5^. Thus far, few studies have evaluated the prognostic significance of elevated transaminases and other hepatobiliary laboratory data for clinical outcomes such as in-hospital mortality and need for ICU admission in Covid-19. Additionally, although SARS-CoV-2 viral RNA has been isolated from human liver samples^3^, few studies have found evidence of a viral hepatitis.

In this study, we analyzed the relationship between commonly obtained hepatobiliary laboratory markers *at admission* and clinical outcomes for patients admitted to the hospital with the diagnosis of Covid-19 to determine if they were of prognostic significance. We additionally evaluated baseline laboratory data to determine if there was any evidence of liver dysfunction or injury in patients with more severe Covid-19.

## Results

### Baseline population demographics

The sample size consisted of 65 patients. The average age was approximately 57 ± 1.93 years, 58% were male and the average BMI was 32.5 ± 1.11 kg/m^2^. There was no difference in BMI between the non-ICU (31.7 ± 1.20 kg/m^2^) and ICU population (34.1 ± 2.34 kg/m^2^; *p* = 0.37). Eighty-six percent of our population was of minority background: 40% (N = 26) of patients were Black/African American, 46% (N = 30) were Hispanic, 8% (N = 5) were Non-Hispanic/White, and 6% (N = 4) did not identify. The median Charlson co-morbidity index^6^ was 3 (IQR: 1–6). Twenty percent (*N* = 13) reported current or former alcohol use. Fewer people admitted to the ICU reported drinking alcohol (*N* = 2, 9%) relative to those not admitted to the ICU (*N* = 11, 25%). Only six patients (9%) had a previous diagnosis of liver disease; 8 patients (12%) had documented Hepatitis C infection and 2 patients (3%) had Hepatitis B infection. Approximately, 49% (N = 32) of our population presented with gastrointestinal symptoms such as nausea, vomiting and diarrhea. The average length of stay was 10.7 ± 1.13 days. ICU admission was required for 32% (N = 21) patients. Mortality was 14% (N = 9). Laboratory data was available for 92% (*N* = 60) of patients included in the study.

### Hepatobiliary laboratory abnormalities are associated with Covid-19 Disease severity

To understand if commonly obtained hepatobiliary labs during admission can be used as prognostic indicators of severity in Covid-19, we compared patients admitted to those not admitted to the ICU. The odds of needing ICU admission with an elevated AST, ALT or ALP above the ULN at any time during admission were 5.12 (95% CI: 1.55–16.89; *p* = 0.007), 4.71 (95% CI: 1.51–14.69; *p* = 0.01) and 4.12 (95% CI: 1.21–14.06, *p* = 0.02), respectively (Table 1). There was no relationship between elevated TB, INR or hypoalbuminemia and the need for ICU admission (Tables 1, 2). Additionally, patients with hypoalbuminemia at the time of admission were more likely to die, develop at least one episode of hypotension, require intubation or need vasopressors by odds ratios of 6.79 (95% CI: 1.27–36.34; *p* = 0.02), 3.56 (95% CI: 1.14–11.12; *p* = 0.03), 8.25 (95% CI: 1.57–43.41; *p* = 0.01) and 6.79 (95% CI: 1.27–36.34; *p* = 0.01), respectively (Table 2). Additionally, 100% of patients who required hemodialysis (N = 6) versus 34% of those who did not had hypoalbuminemia (*p* = 0.002; Table 2).

**Table 1:** Relationship of elevated hepatobiliary laboratory markers at any time during hospital admission and need for admission to the ICU.

	Admitted to the ICU, count (%)	Not admitted to ICU, count (%)	OR (95% CI)	*p*-value	Reference Range^a^
Sample size	21	39	–	*–*	–
AST > 40U/L	16 (76)	15 (39)	5.12 (1.55–16.89)	0.007*	10–40 U/L
ALT > 40U/L	13 (62)	10 (26)	4.71 (1.51–14.69)	0.01*	10–40 U/L
ALP > 120U/L	9 (43)	6 (15)	4.12 (1.21–14.06)	0.02*	30–120 U/L
TB > 1.0 mg/dL	6 (29)	7 (18)	1.83 (0.52–6.39)	0.34, ns	0.3–1.0 mg/dL
INR > 1.0	10 (77), N = 13	13 (59), *N* = 22	2.31 (0.49–10.82)	0.46, ns	–
Lipase > 140U/L	1 (14), N = 7	0 (0), N = 14	–	–	10–140 U/L

A Fishers Exact test was used to make comparisons, with *p* < 0.05 indicating statistical significance. Data are presented as count (percentage of total sample size, *N*). An *implies a significant result. Sample sizes are indicated next to the data point if different from the main group heading. *ALT* alanine aminotransferase, *ALP* alkaline phosphatase, *AST* aspartate aminotransferase, *CI* confidence interval, *ICU* intensive care unit, *INR* international normalized ratio, *ns* non-significant, *SEM* standard error of the mean, *TB* total bilirubin.

^a^Obtained from the ABIM Laboratory Test Reference Range datasheet, https://www.abim.org/~/media/ABIM%20Public/Files/pdf/exam/laboratory-reference-ranges.p.

**Table 2:** The prognostic significance of hypoalbuminemia as it relates to multiple clinical outcomes of Covid-19 disease severity.

	Outcome occurred and had hypoalbuminemia, count (%)	Outcome did not occur and had hypoalbuminemia, count (%)	Odds ratio (95% CI)	*p*-value
ICU admission	10 (48), N = 21	14 (37), N = 38	1.56 (0.53–4.59)	0.58, ns
In-hospital mortality	7 (78), N = 9	17 (34), N = 50	6.79 (1.27–36.34)	0.02*
AKI	12 (46), N = 26	11 (34), N = 32	1.64 (0.57–4.73)	0.42, ns
Need Supplementla oxygen	22 (46), N = 48	2 (18), N = 11	3.81 (0.74–19.51)	0.17, ns
Hypotension	18 (53), N = 34	6 (24), N = 25	3.56 (1.14–11.12)	0.03*
ICU stay > 7 days	7 (64), N = 11	17 (35), N = 48	3.19 (0.82–12.48)	0.09, ns
Need for intubation	8 (80), N = 10	16 (33), N = 49	8.25 (1.57–43.41)	0.01*
Need for vasopressors	7 (78), N = 9	17 (34), N = 50	6.79 (1.27–36.34)	0.02*
Need for hemodialysis	6 (100), N = 6	18 (34), N = 53	–	0.002*

Fishers Exact Test was used to make comparisons between groups, with a *p* < 0.05 indicating significance. Data presented as counts of total population, *N*, with the respecitve outcome; percentages indicate those who had hypoalbuminemia of total population who did or did not have outcome. *AKI* acute kidney injury, *CI* confidence interval, *ICU* intensive care unit, *ns* non-significant.

There was no association between any of the other laboratory parameters and mortality (Supplemental Table 1), development of an Acute Kidney Injury (Supplemental Table 2), hypotension (Supplemental Table 3) or need for supplemental oxygen (Supplemental Table 4). There was additionally no increased odds of ICU admission, prolonged stay in the ICU, in hospital mortality, development of an AKI or hypotension, or need for supplemental oxygen, intubation, vasopressors or hemodialysis based on admission AST, ALT, TB and ALP data above the ULN (Supplemental Table 5, Supplemental Table 6).

### Patient with severe Covid-19 disease have evidence of liver injury

To determine if severe Covid-19 disease is associated with hepatobiliary dysfunction during admission, we compared the changes in AST, ALT, ALP, TB, International Normalized Ratio (INR) and serum albumin to baseline (prior to admission) values in patients based on admission to the ICU (Table 3). Patients admitted to the ICU were found to have higher peak serum concentration of AST (66.5 ± 11.26 U/L, *N* = 18) compared to those not admitted (36.7 ± 3.57, *N* = 34; *p* = 0.01), a greater change in AST concentration from baseline (ICU: 54.2 ± 15.70, *N* = 12; non-ICU: 9.2 ± 4.89, *N* = 24; *p* = 0.01) and a higher peak ALP concentration (ICU: 114.9 ± 13.56 U/L, *N* = 18; non-ICU: 82.3 ± 7.14, *N* = 34; *p* = 0.04). Of note, two patients developed acute liver injury outlined by previously defined criteria^7^ and four had elevated transaminases exceeding > 10 times the ULN.

**Table 3:** The relationship between laboratory data collected at different time points and need for ICU admission during hospital stay.

	Admitted to the ICU, mean ± SEM	Not admitted to ICU, mean ± SEM	*p*-value	Reference range^a^
Sample size^b^, *N*	18	38	–	–
AST, admission, (U/L)	39.1 ± 7.33	34.2 ± 3.68	0.55, ns	10–40 U/L
AST, peak, (U/L)	66.5 ± 11.26	36.7 ± 3.57, *N* = 34	0.01*	
Change in AST, (U/L)	54.2 ± 15.70, *N* = 12	9.2 ± 4.89, *N* = 24	0.01*	
ALT, admission, (U/L)	25.9 ± 3.64	25.0 ± 2.48	0.84, ns	10–40 U/L
ALT, peak, (U/L)	41.4 ± 5.28	29.6 ± 3.35, *N* = 35	0.06, ns	
Change in ALT, (U/L)	13.2 ± 5.70, *N* = 12	4.4 ± 4.49, *N* = 27	0.2, ns	
TB, admission, (mg/dL)	0.7 ± 0.08	0.7 ± 0.08	0.75, ns	0.3–1.0 mg/dL
TB, peak, (mg/dL)	0.8 ± 0.10	0.7 ± 0.06, *N* = 34	0.19, ns	
Change in TB, (mg/dL)	0.2 ± 0.08, *N* = 12	0.1 ± 0.06, *N* = 24	0.65, ns	
ALP, admission, (U/L)	85.5 ± 10.08	80.1 ± 5.59	0.64, ns	30–120 U/L
ALP, peak, (U/L)	114.9 ± 13.56	82.3 ± 7.14, *N* = 34	0.04*	
Change in ALP, (U/L)	36.3 ± 16.92, *N* = 12	0.2 ± 5.12, *N* = 25	0.06, ns	
Albumin, (g/dL)	3.3 ± 0.14	3.6 ± 0.07, *N* = 37	0.10, ns	3.5–5.5 g/dL
INR	1.1 ± 0.04, *N* = 11	1.2 ± 0.12, *N* = 21	0.44, ns	–
Lipase	41.6 ± 18.18, N = 7	39.21 ± 7.65, N = 14	0.90	10–140U/L

Data are presented as mean ± SEM. A Welchs two sample t-test was used to make comparisons with *p* < 0.05 indicating statistical significance. An * implies a significant result. Sample sizes are indicated next to data point if different from main group heading. *ALT* alanine aminotransferase, *ALP* alkaline phosphatase, *AST* aspartate aminotransferase, *CI* confidence interval, *ICU* intensive care unit, *INR* international normalized ratio, *ns* non-significant, *SEM* standard error of the mean, *TB* total bilirubin.

^a^Obtained from the ABIM Laboratory Test Reference Range datasheet, https://www.abim.org/~/media/ABIM%20Public/Files/pdf/exam/laboratory-reference-range;

^b^Four patients (3 in ICU group, 1 in non-ICU group) were excluded from analysis because of elevated transaminase exceeding > 5X the upper limit of normal and were determined to be outliers.

## Discussion

Given the relative novelty of Covid-19, prognostic information is needed to help predict disease severity. In this study we find that the presence of having an abnormal elevation of AST, ALT or ALP during admission increases the odds of admission to the ICU, and thus having more severe disease. Additionally, there was evidence of hepatocellular injury and cholestasis, with an increase in AST and ALP during admission that was worse in patients admitted to the ICU. Elevations in AST and ALT in Covid-19 have been reported by many groups^1,4,5,8^ with AST abnormalities found to be more frequent than ALT abnormalities. It is possible that the presence of underlying co-morbidities such as hepatitis, pre-existing liver disease such as Non-Alcoholic Steatohepatitis (NASH) or alcoholic liver disease predispose patients to develop more severe disease; however, we found no difference in BMI comparing severe (needing ICU admission) vs non-severe disease (no ICU admission), and there were relatively few patients with Hepatitis B or C infection, underlying liver disease or who reported alcohol use in this study. Additionally, given the recent isolation of SARS-CoV-2 viral RNA from human liver samples^3^, it is probable that this virus infects liver tissue. Thus, it appears possible that SARS-CoV-2 can cause viral hepatitis and that this infection can increase disease severity, especially given the association of these markers with the need for ICU admission found in this study. It should be noted that ALT is known to be a more specific marker of hepatocyte injury relative to AST, which is found in many other tissues^9^; thus it is possible that these elevations represent other manifestations of systemic illness. Future studies should examine the incidence of outcomes such as acute liver failure, in Covid-19 patients to understand the relationship between transaminase elevation and clinical outcomes in Covid-19.

Regarding cholestatic liver function, it is not surprising that peak ALP was found to be higher in patients admitted to the ICU and that there was a non-significant trend towards a greater frequency of TB and INR abnormalities in those who died. It is known that cholestatic function can be compromised in critical illness, which can manifest as elevations in ALP and TB^10,11^. Although we did not examine patients for causes of cholestasis in this study, future projects should further characterize these cholestasis abnormalities seen in more severely ill Covid-19 patients.

There may be evidence of mild pancreatic injury in patients with severe covid-19 illness^12^. We did not find any differences in lipase based on ICU admission; furthermore, we found only one patient who had a lipase above the ULN and this data point was < 2X the ULN. Indeed, our sample size was small (N = 21), and neither amylase nor CT-imaging findings were analyzed in this study. However, in line with a recently published study^13^, this data suggests pancreatic injury is not occurring in Covid-19 illness.

We additionally find here that patients with hypoalbuminemia have a higher mortality rate and an association with hypotension in hospitalized patients. Similar findings have already been published by multiple groups^14^, however, most of this data has been studied in the Chinese population. In the USA, it appears that patients of minority backgrounds are disproportionately affected by this illness, and studies to date may not have fully captured this population^15^. Eighty six percent of patients included in this study were of minority background; thus, it appears that the association of hypoalbuminemia and mortality is consistent across multiple populations. Regarding hypotension, albumin has long been known to be the primary driver of oncotic pressure in vascular tissue and hypoalbuminemia is known to cause fluid extravasation and edema; thus, the finding of an association here between hypoalbuminemia and hypotension is not surprising. Future studies should address the influence of ethnicity and background to determine if there are prognostic factors in Covid-19 unique to distinct populations.

The strengths of this study include a focus on minority populations and reliance on objective, easily obtained, biochemical markers to define prognosis in Covid-19. Limitations include a smaller relative sample size, focus on one hospital system and reliance on patient self-report for some data points. Additionally, no data was collected on treatment *or use of medications like acetaminophen, which* may cause elevated transaminases. In summary, we find that elevated transaminases, hypoalbuminemia and other hepatobiliary laboratory abnormalities are associated with a more severe disease course such as need for ICU admission and in hospital mortality and thus may serve as prognostic factors of disease severity in Covid-19.

## Methods

This is a retrospective single-institution case review study conducted in patients admitted with Covid-19 to an academic county hospital in Houston, Texas, USA. Institutional Review Board approval was granted by the University of Texas Health Science Center at Houston, Houston, TX, USA. All research was performed in accordance with relevant guidelines and regulations of Institutional Review Board approval was granted by the University of Texas Health Science Center at Houston, Houston, TX, USA. Patient information was obtained from an electronic medical record system. Patients were included if they were admitted to the hospital after March 1st, 2020 and discharged prior to May 15th, 2020, tested positive for SARS-CoV-2 by polymerase chain reaction (PCR) based testing at least once during admission or if they had a positive diagnosis as an outpatient, and were ≥ 18 years old. Informed consents were waived by Institutional review board at University of Texas Health Science Center at Houston, Houston, TX, USA. The study was conducted in accordance with ethical principles of medical research as noted in declaration of Helsinki.

The primary outcome was to determine the need for ICU admission based on an elevation in Alanine Aminotransferase (ALT), Aspartate Aminotransferase (AST), Alkaline Phosphatase (ALP) or Total Bilirubin (TB) above the upper limit of normal (ULN). Secondary outcomes included in-hospital mortality, development of an AKI, at least one episode of hypotension, need for supplemental oxygen during admission, need for intubation, vasopressors or hemodialysis and prolonged ICU stay, defined as ICU stay lasting longer than 7 days. Admission laboratory values are the first obtained during the hospital stay, 96% of which was obtained within the first 24hours of admission; peak values refer to those that were the highest obtained during admission. Change variables were calculated by subtracting a baseline laboratory value (if available) from the peak data point. Acute liver failure is defined as previously reported^7^: these criteria include the finding of Total Bilirubin > 2 mg/dL and either an elevation in the transaminases or Alkaline Phosphatase > 2 × the ULN. Acute Kidney Injury (AKI) was defined as a rise in serum creatinine from baseline by > 0.3 mg/dL within 48 h. Need for supplemental oxygen was defined as the need for oxygen concentration above room air at any time during admission. Hypotension was defined as any blood pressure reading during admission < 90 mmHg systolic or < 60 mmHg diastolic.

Data was recorded and analyzed using the computer program Microsoft Excel (Redmond, WA, USA). The computer program R^16^ and accompanying R-studio^17^ (version 1.2.5033, Orange Blossom) were used for statistical computations. For continuous and categorical data, a Welch’s two-sided t-test and a Fisher Exact test were used to make comparisons, respectively. For all analyses, a *p*-value < 0.05 was considered statistically significant. The package epiR^18^ was used in the R-studio software to determine odds ratios. Code is available upon request.

## Supplementary Information

Supplementary Information.
