# Supplementary material for: Elevated transaminases and hypoalbuminemia in Covid-19 are prognostic factors for disease severity
Source: Sci Rep. 2021 May 13;11:10308. doi: 10.1038/s41598-021-89340-y (PMC8119433; doi:10.1038/s41598-021-89340-y)
Supplement: Supplementary file 1 — Supplementary Information. [file 41598_2021_89340_MOESM1_ESM.docx]

|  | **Deceased at discharge, Count (%)** | **Survive to discharge, Count (%)** | **Odds Ratio (95% CI)** | ***p*-value** |
| --- | --- | --- | --- | --- |
| Sample Size | 9 | 51 | - | *-* |
| AST > 40 U/L | 6 (67) | 25 (49) | 2.08 (0.47-9.24) | 0.47, ns |
| ALT > 40 U/L | 4 (44) | 19 (37) | 1.35 (0.32-5.64) | 0.72, ns |
| TB > 1.0 mg/dL | 4 (44) | 9 (18) | 3.73 (0.83-16.71) | 0.09, ns |
| ALP > 120 U/L | 4 (44) | 11 (22) | 2.91 (0.67-12.71) | 0.20, ns |
| INR>1 | 6 (100), *n*=6 | 17(58), *n*=29 | - | 0.07, ns |

**Supplemental Table 1**: In-hospital mortality based on the presence of absence of hepatobiliary laboratory abnormalities. Fishers Exact Test was used to make comparisons between groups, with a *p*<0.05 indicating significance. Data are presented as count (percentage of total sample size). An “ * ” implies a significant result. Sample sizes (*n*) are indicated next to the data point if different from the main group heading Abbrevations: *ALT*=Alanine aminotransferase*; ALP*=Alkaline Phosphatase; *AST*=Aspartate Aminotransferase; *CI*=Confidence Interval; *INR*=International Normalized Ratio; *ns*=non-significant; *TB*=Total Bilirubin.

|  | **AKI present, Count (%)** | **AKI absent, Count (%)** | **Odds Ratio (95% CI)** | ***p*-value** |
| --- | --- | --- | --- | --- |
| Sample Size | 26 | 33 | - | *-* |
| AST > 40 U/L | 15 (58) | 15 (45) | 1.64 (0.58-4.61) | 0.43, ns |
| ALT > 40 U/L | 9 (35) | 13 (36) | 0.81 (0.28-2.37) | 0.78, ns |
| ALP > 120 U/L | 9 (35) | 6 (18) | 2.38 (0.72-7.89) | 0.22, ns |
| TB > 1.0 mg/dL | 7 (27) | 6 (18) | 1.66 (0.48-5.72) | 0.53, ns |
| INR>1 | 10 (77), *n*=13 | 13 (59), *n*=22 | 2.31 (0.49-10.82) | 0.46, ns |

**Supplemental Table 2**: Acute Kidney Injury based on the presence or absence of hepatobiliary laboratory abnormalities. Fishers Exact Test was used to make comparisons between groups, with a *p*<0.05 indicating significance. Data are presented as count (percentage of total sample size). An “ * ” implies a significant result. Sample sizes (*n*) are indicated next to the data point if different from the main group heading. AKI is defined as a rise in serum creatinine from baseline by >0.3mg/dL within 48 hours Abbrevations: *AKI*=Acute Kidney Injury; *ALP*=Alkaline Phosphatase; *AST*=Aspartate Aminotransferase; *CI*=Confidence Interval; *INR*=International Normalized Ratio; *ns*=non-significant; *TB*=Total Bilirubin.

|  | **Hypotension present, Count (%)** | **Hypotension absent, Count (%)** | **Odds Ratio (95% CI)** | ***p*-value** |
| --- | --- | --- | --- | --- |
| Sample size | 35 | 25 | - | *-* |
| AST > 40 U/L | 20 (57) | 11 (44) | 1.70 (0.60-4.78) | 0.43, ns |
| ALT > 40 U/L | 16 (46) | 7 (28) | 2.17 (0.72-6.49) | 0.18, ns |
| ALP > 120 U/L | 11 (31) | 4 (16) | 2.41 (0.67-8.70) | 0.23, ns |
| TB > 1.0 mg/dL | 10 (29) | 3 (12) | 2.93 (0.71-12.04) | 0.20, ns |
| INR>1 | 14 (67), *n*=21 | 9 (64), *n*=14 | 1.11 (0.27-4.60) | 1.0, ns |

**Supplemental Table 3**: Hypotension based on the presence or absence of hepatobiliary laboratory abnormalities. Fishers Exact Test was used to make comparisons between groups, with a *p*<0.05 indicating significance. Data are presented as count (percentage of total sample size). An “ * ” implies a significant result. Sample sizes (*n*) are indicated next to the data point if different from the main group heading. Hypotension is defined as any blood pressure reading during admission <90 mmHg systolic or <60 mmHg diastolic. Abbrevations: *ALT*=Alanine aminotransferase*; ALP*=Alkaline Phosphatase; *AST*=Aspartate Aminotransferase; *CI*=Confidence Interval; *INR*=International Normalized Ratio; *ns*=non-significant; *TB*=Total Bilirubin.

|  | **Need for supplemental oxygen, Count (%)** | **Did not need supplemental oxygen, Count (%)** | **Odds Ratio (95% CI)** | ***p*-value** |
| --- | --- | --- | --- | --- |
| Sample Size | 48 | 12 | - | *-* |
| AST > 40 U/L | 26 (54) | 5 (42) | 1.65 (0.46-5.95) | 0.52, ns |
| ALT > 40 U/L | 21 (44) | 2 (17) | 3.89 (0.77-19.69) | 0.10, ns |
| ALP > 120 U/L | 13 (27) | 2 (17) | 1.86 (0.36-9.63) | 0.71, ns |
| TB > 1.0 mg/dL | 11 (23) | 2 (17) | 1.49 (0.28-7.82) | 1.0, ns |
| Albumin<3.5g/dL | 22 (48), *n*=46 | 2 (18), *n*=11 | 3.81 (0.74-19.51) | 0.17, ns |
| INR>1 | 18 (62), *n*=29 | 5 (83), *n*=6 | 0.33 (0.03-3.18) | 0.63, ns |

**Supplemental Table 4** : Need for supplemental oxygen based on the presence or absence of hepatobiliary laboratory abnormalities. Fishers Exact Test was used to make comparisons between groups, with a *p*<0.05 indicating significance. Data are presented as count (percentage of total sample size). An “ * ” implies a significant result. Sample sizes (*n*) are indicated next to the data point if different from the main group heading Need for supplemental oxygen is defined as need for oxygen concentration above room air at any time during admission. Abbrevations: *ALT*=Alanine aminotransferase*; ALP*=Alkaline Phosphatase; *AST*=Aspartate Aminotransferase; *CI*=Confidence Interval; *INR*=International Normalized Ratio; *ns*=non-significant; *TB*=Total Bilirubin.

|  | **Admission to the ICU, OR (95% CI)** | **Moratality, OR (95% CI)** | **Develop AKI, OR (95% CI)** | **Develop Hypotension, OR (95% CI)** | **Need for supplemental oxygen, OR (95% CI)** |
| --- | --- | --- | --- | --- | --- |
| AST > 40 U/L | 1.16 (0.35, 3.81) | 0.76 (0.14, 4.08) | 1.15 (0.35, 3.73) | 0.89 (0.28, 2.83) | 0.67 (0.17, 2.61) |
| ALT > 40 U/L | 0.92 (0.20, 4.11) | 0.67 (0.07, 6.13) | 0.59 (0.13, 2.61) | 0.52 (0.12, 2.16) | 0.85 (0.15, 4.75) |
| TB > 1.0 mg/dL | 1.13 (0.24, 5.29) | 2.14 (0.36, 12.80) | 1.32 (0.30, 5.86) | 1.22 (0.26, 5.66) | 0.71 (0.13, 4.08) |
| ALP > 120 U/L | 1.29 (0.32, 5.22) | - | 0.82 (0.20, 3.27) | 1.09 (0.27, 4.34) | 1.00 (0.18, 5.46) |

**Supplemental Table 5:** The prognostic value of hepatobiliary admission (collected within 24hours of admission) data on clinical outcomes during hospitalization. Fishers Exact Test was used to make comparisons between groups, with a *p*<0.05 indicating significance Data presented as odds ratios (95% confidence interval). An “ * ” implies a significant result. Abbrevations: *ALT*=Alanine aminotransferase*; ALP*=Alkaline Phosphatase; *AST*=Aspartate Aminotransferase; *CI*=Confidence Interval; *OR=*Odds Ratio; *TB*=Total Bilirubin.

|  | **ICU stay >7 days** | **Need for intubation, OR (95% CI)** | **Need for vasopressors, OR (95% CI)** | **Need for hemodialysis, OR (95% CI)** |
| --- | --- | --- | --- | --- |
| AST > 40 U/L | 1.04 (0.24, 4.52) | 0.26 (0.03, 2.23) | 0.30 (0.03, 2.61) | - |
| ALT > 40 U/L | 1.33 (0.24, 7.51) | - | 0.67 (0.07, 6.13) | - |
| ALP > 120 U/L | 0.44 (0.05, 3.93) | - | 0.58 (0.06, 5.26) | - |
| TB > 1.0 mg/dL | 1.59 (0.28, 9.20) | 0.68 (0.07, 6.25) | - | - |

**Supplemental Table 6**: The prognostic value of hepatobiliary admission data on clinical outcomes during hospitalization. Fishers Exact Test was used to make comparisons between groups, with a *p*<0.05 indicating significance. Data presented as odds ratios (95% confidence interval). Abbrevations: *ALT*=Alanine aminotransferase*; ALP*=Alkaline Phosphatase; *AST*=Aspartate Aminotransferase; *CI*=Confidence Interval; *OR*=Odds Ratio; *TB*=Total Bilirubin.

­­­
